# New-Onset Ulcerative Colitis in a Young Caucasian Woman with Unclassified Arteritis

**DOI:** 10.1155/2022/7773222

**Published:** 2022-01-07

**Authors:** Bryan Roberts

**Affiliations:** Kirksville College of Osteopathic Medicine, A.T. Still University, Kirksville, Missouri, USA

## Abstract

Takayasu arteritis is a rare disease mostly found in Asian populations. Cases have been reported in patients with inflammatory bowel disease, suggesting possible genetic linkage. The objective of this clinical case report is to highlight a rare finding of arteritis signs and symptoms in a 32-year-old Caucasian woman (likely early that it did not yet meet classification for official diagnosis as Takayasu arteritis) who subsequently was diagnosed with ulcerative colitis a few months later. The patient presented to the hospital with throbbing neck pain and tenderness around the area of her right carotid artery distribution, nonspecific visual changes, and bilateral upper extremity paresthesia, with significant findings of 50-69% right carotid artery stenosis on a recent outpatient carotid Doppler ultrasound. Based on additional laboratory, clinical, and advanced imaging findings at the hospital, a diagnosis of arteritis not yet classifiable as Takayasu arteritis was made, and the patient was treated with corticosteroids. Unfortunately, she developed bradycardia that was later attributed to the corticosteroid regimen and the medication was discontinued. By follow-up in the clinic, the patient's carotidynia improved, but now, she reported a three-month history of bloody stools. Colonoscopy and pathology findings were consistent with ulcerative colitis, and the patient was started on mesalamine. The association of inflammatory bowel disease and Takayasu arteritis should not be overlooked, as future treatment methods and early, continuous surveillance may be critical in improving quality of life and avoiding serious complications.

## 1. Introduction

Takayasu arteritis is a rare form of vasculitis that is defined by inflammation of the aorta and its large branches [1]. The disease process can be asymptomatic in some cases but otherwise can have a heterogeneous distribution of symptoms [2]. Symptoms can involve neurological, cardiologic/vascular, constitutional, or dermatological manifestations [2]. Of note, symptoms may include general achiness, fatigue, or tenderness of the areas surrounding the inflamed arteries [3]. In more serious cases, this chronic inflammation leads to narrowing of the major arteries, subsequent claudication, or abnormal blood pressure differences between the extremities, visual disturbances, and stroke [4]. Significantly, the erythrocyte sedimentation rate (ESR) is a commonly elevated acute phase reactant in this rare vasculitis [5]. The etiology of the disease is still unclear, but it is commonly found in patients assigned female at birth of Asian descent [6]. Of recent interest, there appears to be a genetic similarity between this disease and another, much more common autoimmune disease, inflammatory bowel disease [7]. Previous case studies have shown similar human leukocyte antigen (HLA) B52, HLA-DR2, and HLA-B39 haplotypes in patients with coexisting Takayasu arteritis and ulcerative colitis [8–11]. Within the past year, analyses of whole genomes, diseases, and traits, including thorough analyses comparing hundreds of thousands of genetic markers with Takayasu arteritis patients, are showing the closest similarity in genetic signatures with inflammatory bowel disease [7].

In the following case, we present a 32-year-old Caucasian female with throbbing neck pain and tenderness around the area of the right carotid artery distribution and elevated ESR who was diagnosed with arteritis not yet classifiable as Takayasu arteritis. While treated with the standard treatment of methylprednisolone, her symptoms improved but she developed episodes of bradycardia, later determined to be a side effect of the methylprednisolone. On follow-up, the patient presented with complaints of bloody stools which was not mentioned during her hospital stay. After gastroenterology workup including colonoscopy and biopsy, she was diagnosed with ulcerative colitis and treated with mesalamine.

At a glance, this patient's two diseases (one confirmed and the other not yet classifiable) seemed mutually exclusive, given one disease is quite rare and affects large arteries and another disease is common and affects the gastrointestinal tract. However, given the close genetic similarities of these diseases, it would be important to continue following this patient and others, as new genetic discoveries could lead to more effective and early surveillance and treatment regimens to limit worsening disease progression.

## 2. Case Presentation

### 2.1. History of Present Illness

A 32-year-old Caucasian female initially presented to the hospital from a clinic complaining of right-sided neck pain, nonspecific visual changes, and bilateral upper extremity paresthesia. The neck pain was present for two weeks, acutely worsening two days prior to evaluation and subsequent hospitalization. The neck pain was throbbing in nature and radiating to the jaw. Her nonspecific visual changes occurred 4 days ago but resolved spontaneously. She denied a history of trauma or falls. She also denied limb claudication. The patient had an outpatient Doppler ultrasound (US) evaluation of her carotids, which showed 50-69% stenosis of her right carotid artery. Labs in the clinic were notable for elevated ESR of 67 mm/hr and elevated C-reactive protein (CRP) of 2.7 mg/dL but were otherwise within normal limits. She was prescribed methylprednisolone 125 mg every six hours and transferred to the hospital for further evaluation.

Her past medical history included anxiety, for which she was taking escitalopram 5 mg daily, and hyperlipidemia, for which she was taking atorvastatin 40 mg nightly. Five years ago, she was seen by a specialist for “lower-end-of-normal” thyroid levels, but her recent thyroid-stimulating hormone (TSH) level was within normal limits. She also reported the history of a massive blood transfusion one year prior when she was diagnosed with placental abruption. The patient's family medical history was significant for coronary artery disease, hyperlipidemia, hypertension, and diabetes. Her surgical history consisted of five cesarean section deliveries and one dilatation and curettage. She had no known drug allergies or seasonal allergies. She was currently working as a nurse. She had no significant alcohol or recreational drug use but was a former cigarette smoker.

### 2.2. Review of Systems

A detailed review of systems revealed a lack of fevers, chills, sweats, headaches, or fatigue. She denied ear pain, nasal congestion, sore throat, cough, or shortness of breath. She reported bilateral numbness in her upper extremity but denied focal weakness. She also denied chest pain, nausea, vomiting, or abdominal pain. She had right-sided neck pain but denied temporal pain or tenderness, joint pain, limb claudication, rashes, pruritus, abrasions, muscle aches, or early morning stiffness. She also denied constipation, diarrhea, dysuria, or hematuria.

### 2.3. Physical Findings

Physical examination revealed an oral temperature of 36.6°C, pulse of 81 beats per minute, respiratory rate of 16 breaths per minute, blood pressure of 109/64 mmHg, and saturation of peripheral oxygen (SpO_2_) of 100% on room air. She was oriented to person, place, time, and situation, and in no acute distress. Pupils were equal, round, and reactive to light, extraocular muscles were intact, and there was no scleral icterus or hyperemic conjunctivae. The neck was supple, with significant tenderness to very light palpation of the right lateral neck area surrounding her right carotid artery distribution, with no contralateral similarity. Additionally, there was no induration, ecchymosis, erythema, or edema of the area of described and elicited discomfort. No lymphadenopathy, thyroid nodules, goiter, or thyroid tenderness were noted. No jugular venous distention was observed, and no carotid bruits were auscultated. Lungs were clear to auscultation bilaterally, and respirations were nonlabored. Heart rate and rhythm were normal, with no significant murmurs or gallops. Pulses were 2/4 in all extremities, with no peripheral edema. Blood pressure measurements of all four limbs were not performed. The abdomen was soft, nontender, and nondistended with normoactive bowel sounds and no masses. The skin was warm, dry, and pink with no significant areas of rashes or ecchymosis. Cranial nerves two through twelve were intact, upper and lower extremity strength was 5/5 bilaterally, and sensations were intact bilaterally. The patient was cooperative with appropriate mood and affect, with no significant confusion or memory loss. A breast exam and gynecologic exam were not performed.

### 2.4. Initial Differential Diagnosis and Initial Plan

Pain and tenderness around the carotid artery distribution can be associated with a range of vascular and nonvascular diagnoses. A significant vascular cause for immediate consideration is arterial dissection. Other vascular causes include the large-vessel vasculitides such as giant cell arteritis and Takayasu arteritis, as well as jugular vein thrombosis and carotid artery stenosis. Nonvascular causes include cervical lymphadenitis, head and neck tumors, and submandibular gland disorders like sialolithiasis or sialadenitis. Also included in the differential are general musculoskeletal causes that result in pain and tenderness on the side of the neck, such as a muscle strain, ligament sprain, or disc herniation. The patient's history did not reveal trauma to the neck region, and clinic Doppler US findings showed vascular turbulence, so it was less likely her symptoms were due to musculoskeletal causes alone.

The initial plan for this patient was to order a battery of labs and imaging studies and request consultation from cardiovascular surgery and rheumatology. Labs ordered were comprehensive as well as purposely focused on the exclusion of rheumatologic etiologies. These included complete blood count with differential (CBC), basic metabolic panel (BMP), CRP high-sensitivity, ESR, and autoimmune markers of rheumatoid factor, antineutrophil antibody (ANA), anti-centromere antibody, anti-DNA antibody, anti-Jo-1 antibody, anti-chromatin antibody, anti-Sc1-70 antibody, anti-ribonucleoprotein antibody, anti-SS-A and anti-SS-B antibody, and anti-Smith antibody. Outpatient carotid Doppler US was notable for 50-69% right carotid artery stenosis, a finding that prompted a carotid computed tomography angiography (CTA), head CTA, and chest magnetic resonance angiography (MRA). These modalities provided a more detailed look at the patient's vascular supply, further determining if there was any evidence of arterial dissections, stenosis, thrombosis, or structural evidence of vasculitides.

The ESR was elevated at 36 mm/hr, and the high-sensitivity CRP was elevated at 0.71 mg/dL, but the other labs were within normal limits. The autoimmune markers ordered were negative. Imaging studies were also negative. Despite significant findings of 50-69% right carotid artery stenosis on prehospitalization carotid Doppler US, the carotid CTA showed 0% stenosis of the extracranial internal carotid arteries using North American Symptomatic Carotid Endarterectomy Trial (NASCET) criteria, no arterial dissections, and no pseudoaneurysms [12]. In addition, there was no significant stenosis or occlusion of the bilateral subclavian arteries or extracranial vertebral arteries. The aortic arch appeared normal, and the origins of the left subclavian artery, left common carotid artery, and innominate artery were also normal. Though the left vertebral artery was shown to arise from the aortic arch, this is a normal variant. The head CTA revealed no acute intracranial abnormalities, no intracranial aneurysm, arteriovenous malformation, arterial dissection, arterial stenosis, or arterial occlusion of the anterior or posterior cerebral circulation. Lastly, the chest MRA demonstrated normal caliber and patency of the aorta, left subclavian artery, left common carotid artery, and innominate artery without evidence of inflammation. Additionally, the heart size was normal, the lungs had normal signal, there was no evidence for pericardial or pleural effusions, and no significant lymphadenopathy was found. Given those unremarkable imaging findings, including no structural evidence of Takayasu arteritis, there was no acute necessity for cardiothoracic surgical intervention at that time.

Rheumatologic focus was maintained due to the persistently elevated ESR and CRP, with early-stage Takayasu arteritis as the working differential. Albeit a lack of radiologic evidence and nontraditional presenting symptoms, not meeting the American College of Rheumatology classification criteria of Takayasu arteritis, an elevated ESR and carotidynia can be appreciated in early stages of the disease process making Takayasu arteritis a continued consideration. The initial treatment was focally anti-inflammatory with administration of methylprednisolone 125 mg every 6 hours and continued monitoring of symptoms.

### 2.5. Final Diagnosis and Intervention

The initial management plan was to treat the patient's inflammation with methylprednisolone, considering Takayasu arteritis given her ongoing right carotidynia and elevated ESR but without the evidence of diagnostic criteria. The patient's right carotidynia exhibited significant improvement during her hospital stay as the corticosteroid regimen took effect; however, she did experience some complications. She was woken up in the middle of the night by a sharp, stabbing pain in her thoracic back and episodes of heaviness in her chest, shortness of breath, and tingling in her right arm. Heart rate monitoring showed 40-50 beats per minute, and SpO_2_ was 94% on room air. After a negative cardiac and endocrine workup including cardiac enzymes, 12-lead electrocardiogram, and normal TSH level, there were no significant acute concerns of myocardial injury, arrhythmias, or myxedema.

Despite limited supportive evidence, the working differential remained Takayasu arteritis without yet meeting official diagnostic criteria. The patient was discharged on prednisone 20 mg daily with follow-up on an outpatient basis, increasing to 60 mg daily if the neck pain returned. When she followed up initially, she noted that she stopped the prednisone because she felt that it was related to her bradycardia episodes. She was no longer having the bradycardia episodes, and no more carotidynia besides a mild episode on her left neck which lasted a couple of days and resolved on its own.

Interestingly, the patient now seemed concerned about bloody diarrhea that had been going on for a few months, a new finding from what she stated during the review of systems throughout her hospital stay. She was experiencing 6-8 loose, slightly bloody bowel movements daily. The bloody bowel movements happened throughout the day, and sometimes she would also pass blood through her rectum when sitting to void. The blood was bright red to maroon. She experienced urgency to have bowel movements that were not prompted by eating. She denied fevers, chills, or abdominal pain.

Given her history of elevated inflammatory markers and new presenting symptoms of bloody diarrhea, she would now be evaluated for inflammatory bowel disease. The patient was tested for perinuclear anti-neutrophil cytoplasmic antibodies (P-ANCA), which came back abnormal at 1 : 640 titer. Positive P-ANCA can be associated with vasculitis such as Takayasu arteritis, but also inflammatory bowel disease. Colonoscopy was promptly ordered to evaluate the patient's bloody stools further. Colonoscopy revealed aphthous ulceration and friable mucosa of the descending colon, along with a sessile 5 mm rectal polyp. The terminal ileum did not show signs of gross inflammation or abnormalities. Biopsy was performed on the descending colon mucosa, and the rectal polyp was removed, with both sent for pathology review. Stool aspirate was also taken during the colonoscopy to perform a multiplex polymerase chain reaction (mxPCR) stool pathogen analysis. Pathology review of the descending colon biopsy confirmed chronic inflammatory bowel disease with mild activity most consistent with ulcerative colitis, and the mxPCR stool pathogen panel showed positive results only for *Campylobacter*. All biopsies were negative for dysplastic or malignant changes.

### 2.6. Follow-Up and Outcomes

Management after diagnosis of ulcerative colitis and infection with *Campylobacter* included prompt antibiotic use with ciprofloxacin and follow-up with gastroenterology. On follow-up with gastroenterology, the patient had completed her course of antibiotics and her symptoms felt approximately 50% better; however, she was still passing bright red blood in her stools with about 3-4 bowel movements daily. Repeat CRP high-sensitivity was elevated at 1.114 mg/dL. She was prescribed mesalamine 4.8 g daily for treatment of the colitis and would need to follow up with gastroenterology for medication management in the future. She did not report having the carotidynia, but it would be important to continue monitoring for those episodes as well. If the pain episodes would recur, alternatives to prednisone would be considered since the patient likely had an adverse reaction to methylprednisolone and prednisone during her hospital stay and subsequent outpatient treatment. Alternatives to discuss with her included methotrexate and azathioprine, which also coincidentally could be used to treat ulcerative colitis.

## 3. Discussion

On the surface, Takayasu arteritis and ulcerative colitis are two autoimmune disorders that do not seem interrelated. Takayasu arteritis is a rare chronic autoimmune disease of the aorta and its main branches such as the carotid arteries [8]. It is considered a rare disease most likely found in Asian populations but can occur worldwide [6]. Patients assigned female at birth are more likely to have this disorder than those assigned male at birth [6]. Age of diagnosis most commonly happens under forty years old [13]. Ulcerative colitis is a common chronic autoimmune disease of the large intestine [14]. Caucasian individuals of westernized societies are most likely to be affected by this disease [15]. It affects the female and male biological sexes about evenly [15]. Ulcerative colitis has a bimodal age distribution of diagnosis, between late teens and twenties or later than fifty years old [16].

However, as this case helps to present, these two disorders can coexist. The evidence for coexistence of Takayasu arteritis and ulcerative colitis is still rare, but reports of patients with these two diseases simultaneously continue to develop [17]. The coexistence of Takayasu arteritis and ulcerative colitis has led researchers to find causal genetic linkages. Initially, researchers found these patients with coexisting disease had similar HLA-B52 and DR2 haplotypes [8]. Other studies found promise in the link of the HLA-B39 haplotype [9]. Most importantly, these genetic haplotypes seemed to be found most commonly in Asian individuals and quite rarely in Caucasian individuals [8, 9].

It is possible for Caucasian individuals to carry the HLA-B52 and DR2 haplotypes, and when they do, they seem to have the simultaneous coexistence of Takayasu arteritis and ulcerative colitis [10]. One case report gives an example of a 37-year-old non-Asian assigned female at birth with the HLA-B52 and DR2 haplotype who was first diagnosed with Takayasu arteritis and subsequently developed ulcerative colitis [10]. When she was treated with mesalamine, the ulcerative colitis would not be controlled, and she needed to continue steroids [10]. However, when azathioprine was administered, both her ulcerative colitis and Takayasu arteritis went into remission [10].

One decade later, other researchers continued to find HLA-B52 genotype similarities in patients with both diseases [11]. These researchers considered ulcerative colitis as a complication of Takayasu arteritis [11]. It would be important in those cases, such as the case presented in this paper, to offer close surveillance of these patients with Takayasu arteritis for symptoms such as abdominal pains and hematochezia or other stool abnormalities to offer prompt diagnostic tests for ulcerative colitis. Other studies suggest that if patients being treated with ulcerative colitis start having symptoms of Takayasu arteritis such as carotidynia, hypertension, fever, and continued elevated acute phase reactants like ESR and CRP, it would be important to promptly evaluate for this disease as well [18].

Unfortunately, the previous studies were still small and did not involve a comprehensive variety of individuals. This knowledge changed in the past year with promising results from a large genome-wide study [7]. Most recently, the largest study to date involving over 6670 individuals including over 1200 with Takayasu arteritis within different global populations determined critical insight about patient susceptibility to contracting Takayasu arteritis [7]. Of hundreds of traits compared, whole-genome analysis, and genetic marker analysis, the researchers found the closest genetic similarity between Takayasu arteritis and inflammatory bowel disease [7]. It is becoming clearer that these seemingly unrelated diseases have meaningful close associations. Thus, this significant contribution could lead to new options for treatment and diagnosis or repurposing of current treatments for these diseases.

Given Takayasu arteritis is a rare and still unfamiliar disease, its significant genetic connection to inflammatory bowel disease should continue to be evaluated. Particularly in cases such as the one presented in our study, where the patient may be unable to tolerate common treatments for one disease, other treatment options could be pursued for a more individualized approach to coexisting or even single disease. It may be important in cases such as this patient where the disease is not showing significant vascular findings, to continue surveillance to be proactive in the patient's treatment and avoid serious complications.

## Data Availability

The data is available from the author upon reasonable request.
